# A breath of fresh air: a quality-improvement study comparing an air-circulating technique versus conventional technique to prevent nasogastric tube dysfunction

**DOI:** 10.1186/s12912-015-0111-9

**Published:** 2015-11-27

**Authors:** Murad Bani Hani, Ikenna Ihim, Joyce Harps, Steven C. Cunningham

**Affiliations:** The Department of Surgery, Saint Agnes Hospital, Baltimore, MD USA; The Department of Nursing, Saint Agnes Hospital, Baltimore, MD USA; Pancreatic and Hepatobiliary Surgery, Saint Agnes Hospital, 900 Caton Avenue, MB 207, Baltimore, MD 21229 USA

**Keywords:** NGT, Nasogastric tube, Aspiration, Pneumonia, Patient safety

## Abstract

**Background:**

Nasogastric tubes are an important component of care in patients with gastrointestinal obstructions. However, they are prone to malfunction despite conventional flushing techniques, with potentially severe consequences. There is no widely accepted, gold-standard way to ensure that a nasogastric tube succeeds in maintaining an empty stomach following flushing.

**Methods:**

We have developed a flushing technique to better ensure successful tube function. We compared this technique to conventional flushing both in vitro (using a plastic stomach model) and in vivo (in a pig model), and we provide a didactic video.

**Results:**

The mean gastric residual volume following our novel flushing technique is nearly 25-fold lower than following conventional flushing (13 mL vs. 330 mL).

**Conclusions:**

Our simple technique is more effective than conventional techniques in maintaining nasogastric tube function and therefore should prevent dangerous vomiting and aspiration pneumonia better than conventional techniques.

## Background

**Fig. 1:**
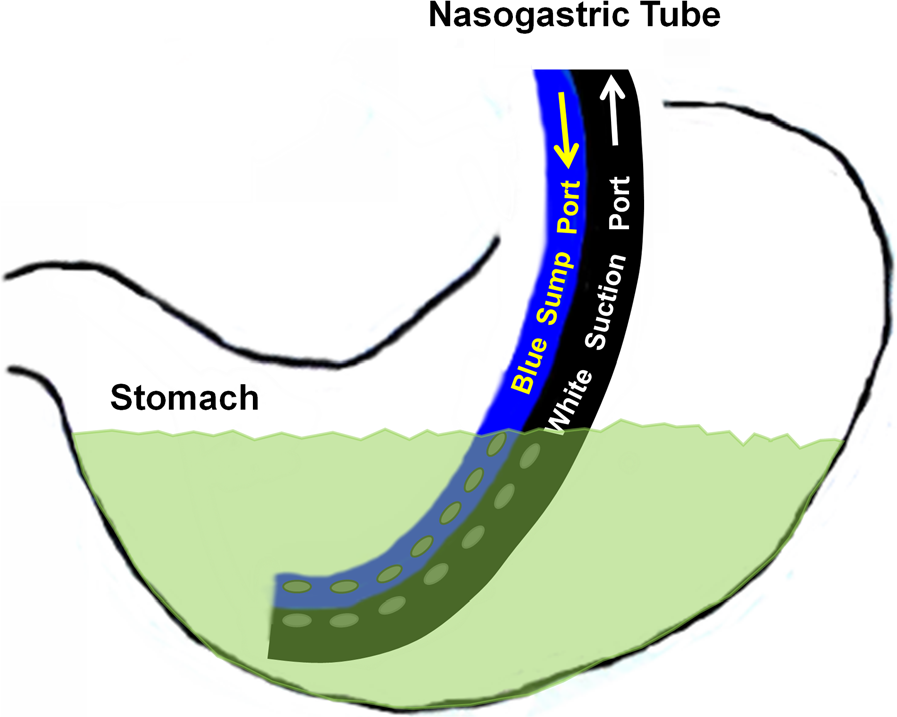
Diagram of a Nasogastric Tube. The modern nasogastric tube is a double-lumen tube, with a main, larger (often white) suction port, and an second, smaller (often blue), air-sump side port

The nasogastric tube (NGT) is used in a wide variety of patients to provide gastric decompression when needed, for example, in cases of gastric-outlet [1–3] or small-bowel obstruction [4] (Fig. 1). Proper functioning of the NGT is important because failure to keep the stomach and proximal small bowel decompressed may increase the risk of vomiting, leading to aspiration pneumonia, a common and potentially life-threatening event [5].

The stomach, however, is poorly suited to empty via an NGT. While larger (14- or 18-F) tubes are more effective at gastric emptying than smaller tubes (10-F) [6], even 16- or 18-F NGTs may fail to empty the stomach reliably and prevent vomiting. This may be due to the slack and mobile mucosal folds that become suctioned onto tube ports, evidenced by suction lesions at endoscopy [7]. Conventional NGT maintenance includes simple flushing, which will push this mucosa away, but suction easily pulls it back into the orifices. Although conventional flushing will help avoid mucous plugging, it does not address the suctioning of gastric mucosa back into the NGT orifices, which may creates a one-way valve, such that fluid may pass into the stomach during conventional flushing, but not out of the stomach when suction is applied. This is why simply replacing the NGT fails to solve the problem, because the problem is not with the tube, it is with the stomach. The NGT must be therefore be assessed for patency and function in both directions, not only antegrade into the stomach, but retrograde out of the stomach.

We hypothesized that a flushing technique that ensures bidirectional patency and function of the NGT would more effectively ensure an empty stomach. The basis of the new technique is the fact that, while suction is applied to the main port, air injected into the side port should return immediately out the suction port of an appropriately placed NGT if and only if the stomach is empty. Accordingly, the observation of air circulating out the suction tubing immediately after being injected in the blue side port is a key indicator of bidirectional patency and function. By contrast, air injected into the side port would not be expected to suction out of the main port if the end of the NGT is submerged in a pool of gastric fluid, because the air would simply bubble to the top of the pool (Figs. 1, 4 and 5). We have designed a new flushing technique that relies on this visualization of flushed air to circulate immediately out the suction tubing.

When an extensive literature search failed to locate such a technique, we proceeded to assess our new flushing technique, comparing it to the conventional technique taught in nursing textbooks. Since vomiting is well recognized to occur in patients even following “successful” conventional NGT flushing, a better way to ensure an empty stomach is essential.

## Methods

### Literature search

The lack of guiding literature on the best way to ensure proper function of an NGT for gastric decompression is well recognized [6, 7]. Current evidence-based recommendations to prevent aspiration pneumonia include maintaining the head of the bed at 30–45 degrees and ensuring proper NGT placement [8–10], or entirely redesigning the NGT [11, 12], but there is little guidance regarding assessing the NGT as the suction device in current widespread use. Evidence-based reviews suggest checking gastric residual volume (GRV) (defined as the volume left in the stomach after flushing an NGT) during gastric feeding [10, 13, 14] but do not specifically address how best to assess GRV, nor how to ensure that the measured GRV is accurate.

Therefore, a comprehensive literature search was conducted using PubMed and the inclusive keywords “nasogastric tube” with the following terms: flush (14), irrigation (74), suction (137), maintenance (61), care (1134), function (1080); aspiration pneumonia (186), residual (62), small-bowel obstruction (92), sump (15). The resultant list of 2855 citations contained 2214 unique citations. An additional search of simply “nasogastic tube” was performed to avoid missing any relevant citations and returned 3970 results, which were limited to adults, to the English language, to human studies, and to studies containing “nasogastric tube” in the title, leaving 325 citations. These 325 titles were independently scanned by two authors (MHB and SCC) for relevance regarding the care and maintenance of the NGT, the checking of gastric residuals, and the prevention of aspiration pneumonia. Relevant papers were then reviewed and manual cross-referencing was performed using each article’s reference list, to identify all published data about NGT function and care, which revealed no additional relevant studies.

### In vitro experiments

An in vitro model of the stomach was designed using floppy plastic bags (grocery bags) modeling the floppy gastric mucosa, placed within 500-mL rigid plastic canisters modeling the more rigid gastric wall. This in-vitro model of the stomach was filled with warm tap water and an NGT was placed into the water with the end of the NGT in the most dependent portion.

Suction was applied to the NGT at -40 mm Hg, and the water was allowed to aspirate until a point at which either the model stomach was empty or fluid ceased to aspirate out (and remained ceased for 5 min), suggesting a blockage. The aspiration process was closely inspected for signs of blockage. If the plastic bag was seen to suction into the NGT holes then this was recorded as the reason for blockage (this occurred every time). If this was not seen, then the NGT was disconnected from suction tubing to ensure that there was still suction present (this latter case never occurred). Once this point was reached, the two flushing techniques (the conventional versus the new, air-circulating techniques) were employed as described below. Suction was then reapplied and the water was allowed to aspirate again, until a point at which either the modeled stomach was empty or fluid ceased to aspirate out (and remained ceased for 5 min), suggesting a recurrent blockage. The trial was then terminated and the volume of fluid remaining was measured as the GRV. Experiments were done in triplicate.

To validate that the plastic bag would indeed model the musoca of the human stomach, this model was compared to a similar set-up with the same plastic canister but without the plastic bag. The same volume of water, placement of NGT, flushing and aspiration were used.

### In vivo experiments

With approval of the Johns Hopkins Animal Care and Use Committee and in accordance with the Animal Welfare Act, healthy swine (*Sus domesticus*) underwent laparoscopy followed by laparotomy during a Covidien-sponsored educational lab at the Johns Hopkins Minimally Invasive Surgical Training and Innovation Center, then were euthanized as planned. An NGT was immediately inserted through the mouth and into the stomach, where its position was confirmed by palpation through the stomach wall. Pigs whose stomachs were not empty, or were excessively distorted by the educational lab procedures, were excluded. A single pig was used, approximately 55 lbs, 4 months old, and female.

As an in vivo model of the human stomach, the pig stomachs were filled by instilling warm tap water via the NGT and suction was applied to the NGT at -40 mm Hg. The water was allowed to aspirate until a point at which either the stomach was empty or fluid ceased to aspirate out (and remained ceased for 5 min), suggesting a blockage. Once this point was reached, the tubes were flushed according to either the conventional technique or the new, air-circulating technique (see below), following which the trial was terminated and the volume of fluid aspirated was subtracted from the fluid instilled (the initial 500 mL + the amount flushed) to obtain GRV. Experiments were done in triplicate.

NGTs from Covidien (Mansfield, MA) were employed for all studies (“Salem Sump™ Dual Lumen Stomach Tube,” 18 Fr [6.0 mm] x 48" [122 cm]).

### Flushing techniques

The conventional technique: A widely used technique was used as the conventional technique [15] to maintain patency of an appropriately placed NGT. The main, suction port was flushed with 30 mL of saline using an Asepto syringe, a GRV was aspirated using the syringe, and then 10 mL air were injected into the small, blue, side port, and finally, suction reconnected.

The new, air-circulating technique: A simple, 8-step flushing technique, was designed to overcome the problem of the gastric mucosa occluding the suction orifices. The protocol and rationale for each step is shown in the Table 1. The technique is also demonstrated in the online video "NGT 501" (www.youtube.com/watch?v=VHmQdCTfIzY) [16].

**Table 1:** Eight Steps of the New Flushing Protocol, with Rationale

Step	Rationale
1. During flushing, change suction to continuous. Be sure that the tubing is not clogged.	So that suction does not turn off during the subsequent steps below.
2. Inject 120 mL warm tap water and 120 mL air into larger (clear, suction) port.	So that the stomach lining is pushed away from the tube, and so that there is an adequate volume in the stomach to suction out.
3. Reapply continuous suction; observe and note the character and amount output (Suctioned volumes are not re-administered to the patient).	So that the volume of water injected and gastric fluid already present is suctioned out. Sometimes, repeating step 2 is necessary to get this volume to suction out.
4. Flush 60 mL air into smaller (blue or clear, air-sump) port and watch for air to suction out larger (clear, suction) port.	If the NGT is in an empty stomach, the air injected in the small port will enter the stomach, suction out the suction port, and be seen as large bubbles of air in the suction tubing, but if the NGT is at the bottom of a pool of liquid, this will not happen because the air will simply bubble to the top of the gastric pool.
5. May repeat the 60 mL air into smaller (blue or clear, air-sump) port x3.	Sometimes more air is needed; may also repeat step 2 again here.
6. Call ordering physician if air flushed into smaller (blue or clear, air-sump) port is not suctioned out through larger (clear, suction) port.	Because this means that the stomach may be full of liquid and dangerously distended.
7. If air flushed into smaller port is seen to suction out through the larger port, then intake and output may be recorded, and GRV calculated by subtracting flush volume from total volume suctioned (all flushes should be recorded as intake, and all aspirate as output).	If air flushed into smaller port is immediately suctioned through larger port, then the stomach is empty (Fig. 4a).
8. Return suction to low intermittent suction.	Intermittent is better than continuous suction because intermittent lapses in allow the stomach lining is allowed to fall away from the suction holes of the NGT.

*Abbreviations*: *NGT* nasogastric tube, *GRV* gastric residual volume. *NB*: This protocol assumes that the appropriate position of the *NGT* within the stomach has already been confirmed.

### Statistics

All trials were performed a minimum of three times. Data are presented as mean +/- standard deviation. A paired *t*-test was used to test for significance. Two-tailed significance was accepted at *P* <0.05.

## Results

### Literature search

Not a single study was found regarding how to ensure that a GRV detected after NGT flushing is accurate. Several studies distinguished the volume of gastric secretions/flushes from the volume of tube feeding [17–19], but none addressed how to determine that the aspirated GRV was the true GRV.

### In vitro experiments

The in vitro model of the stomach, using a plastic bag to model the gastric mucosa, was initially compared to a similar model without the plastic bag, to validate the modeling of the mucosa. As expected, an NGT placed within a simple canister without the mucosa-modeling bag to interfere with suctioning, evacuated all 500 mL of fluid in each of three trials. When the mucosa-modeling bag was added, however, only 142 mL (+/- 37.6 mL) of the 500 mL was suctioned out before the mucosa-like bag was observed to suction into, and thereby block the NGT orifices (N = 6, including three conventional-flush trials and three new-flush trials), confirming the ability of the bag-in-canister model to mimic the suctioning of mucosa into the NGT orifices.

Following cessation of flow due to blocked NGT orifices (this occurred every time), two flushing techniques were compared, the conventional technique, and the new, air-circulating technique. The volume at which the aspiration ceased due to blocked NGT orifices did not significantly differ (P = 0.7) for the conventional-flush group (133 mL +/- 29 mL) and the new-flush group (150 mL +/- 50 mL), suggesting that subsequent comparisons are valid.

**Fig. 2:**
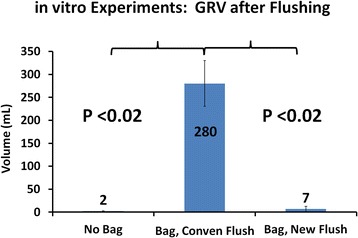
Results of in vitro experiments. See text for details. Conven = Conventional

As shown in Fig. 2, the amount of volume not aspirated from the bag-free canister (the GRV) was negligible. In the canisters with the bag modeling the gastric mucosa, however, most of the 500 mL (280 mL +/- 50 mL) remained in the stomach as a GRV when conventional flushing was used. By contrast, the new, air-circulating technique was significantly more effective at emptying the "stomach," and the GRV returned essentially to nearly zero (Fig. 2, *P* <0.02).

### In vivo experiments

**Fig. 3:**
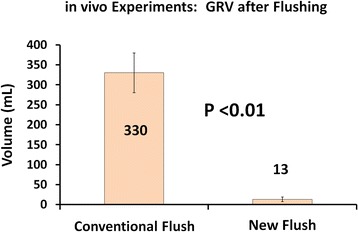
Results of in vivo experiments. See text for details

To confirm these promising results, and to test the efficacy of the new, air-circulating technique in living stomachs, we performed the same comparison in pig stomachs. After instilling 500 mL of warm tap water into the pig stomachs, and applying suction to the NGT, a mean of only 208 mL returned (N = 6, including three conventional-flush trials and three new-flush trials) before flow ceased, presumably due to suctioning of the mucosa into the NGT orifices. The NGT was then flushed with each technique, and the new, air-circulating was significantly more effective at emptying the stomach: After conventional flushing a mean of 330 mL GRV was left behind, but after new, air-circulating flushing, a negligible mean of 13 mL was left behind as the GRV (Fig. 3; *P* <0.01). The volume at which initial aspiration ceased, presumably due to blocked NGT orifices, did not differ (P = 0.5) for the conventional-flush group (150 mL +/-50 mL) and the new-flush group (266 mL +/-208 mL), suggesting that the groups are comparable and the difference seen between the groups is real.

## Discussion

**Fig. 4:**
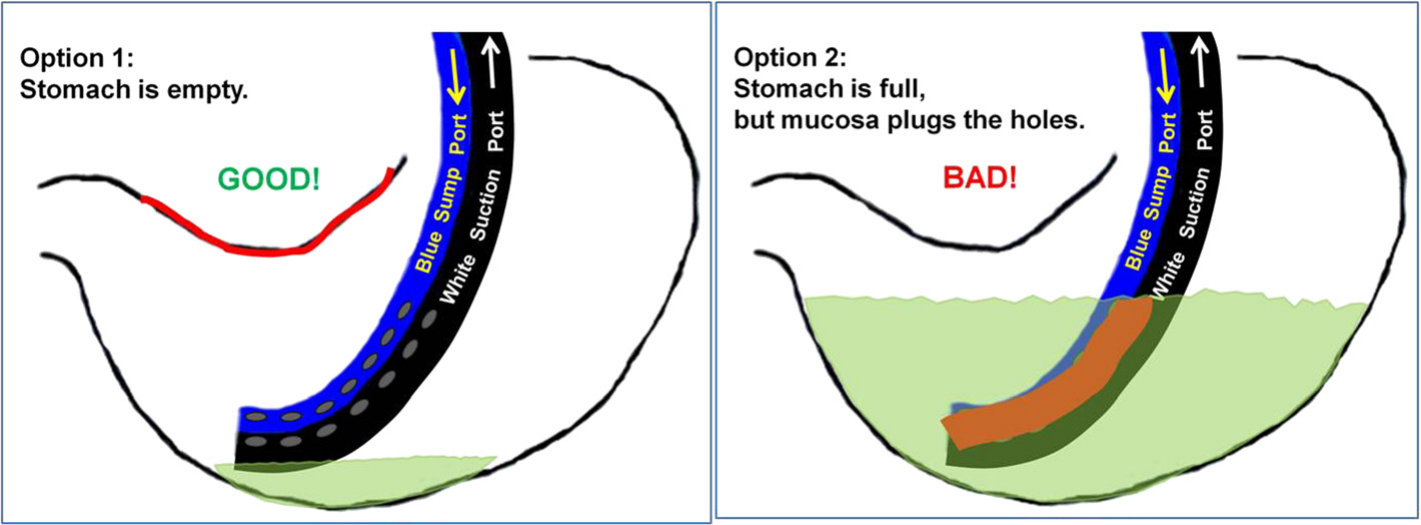
NGT Function and Dysfunction. **a** Option 1: Empty stomach with properly functioning NGT; (**b**) Option 2: Full stomach with dysfunctional NGT. The red lines indicate gastric mucosal lining. When the lining is pulled into the suction holes, the stomach cannot empty

NGTs are known to imperfectly empty the stomach. The consequences of such dysfunction vary from mild to life-threatening, and include nausea, vomiting, and aspiration pneumonia. One common cause of dysfunction is plugging of the lumen (eg, by blood, mucous, or debris) which is easily remedied by conventional flushing. Another common cause is a one-way valve that is produced by gastric mucosa being suctioned onto the NGT orifices. Unfortunately, there is no way in the conventional technique to detect mucosal plugging of the NGT orifices, which might leave undetected a high GRV, or, inversely, fail to confirm an empty stomach. This explains why even well-designed studies on GRV using conventional techniques show that it adds little to the care of patients [20]. When suction is applied after conventional flushing, some quantity of fluid returns, but when this return stops, there is no way to know whether it has stopped because the stomach is empty (Fig. 4a) or because the mucosa has suctioned into the holes, and is blocking them (Fig. 4b).

Following NGT flushing, air is sometimes heard suctioning in through the smaller, air-sump side port, creating a reassuring whistle. This sound demonstrates that the stomach is empty and the side port is patent as an air vent, aspirating air (whistling) from the room through the side port, into the stomach, and then out the suction-port tubing and into the wall. A caveat regarding the whistle is that it is not heard constantly. In fact, if it is heard constantly, the location of the tube should be reassessed because an NGT which has been pulled partially out, and has its tip in the distal esophagus, will sump air more constantly than an NGT correctly placed in the stomach. This occurs because the distal esophageal mucosa is less likely to suction into, and block, the NGT orifices, compared with the more redundant stomach mucosa.

**Fig. 5:**
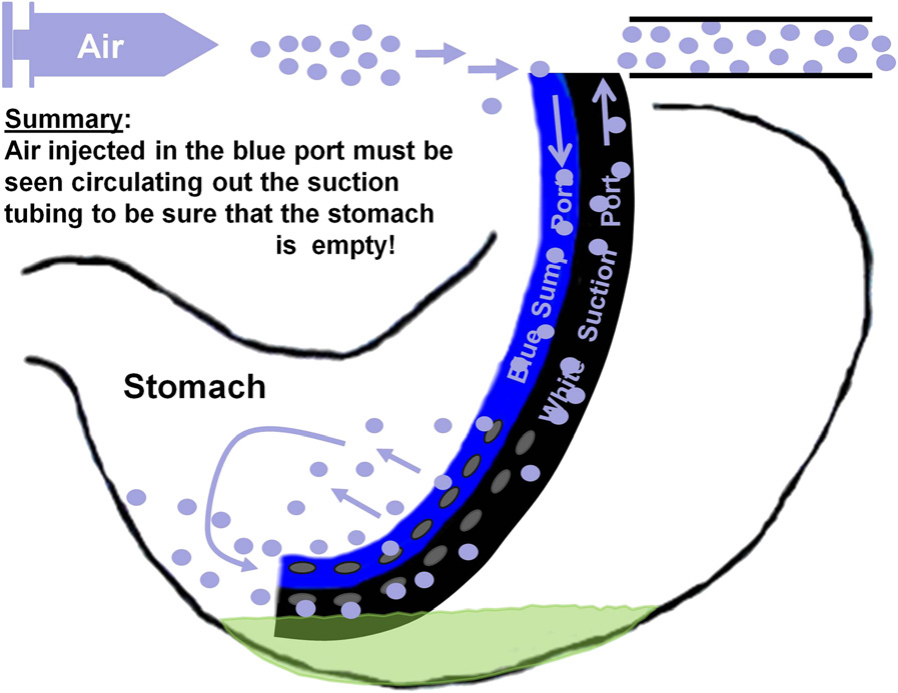
Properly Functioning NGT. While continuous suction is applied to the larger, white, suction port, air injected through the smaller, blue, air-sump port must be seen circulating out the suction tubing to be sure that the stomach is empty. When room air is pulled into the blue air-sump port, a reassuring whistle is sometimes heard. A 4-minute video demonstrating these principles and technique [16] is available at www.youtube.com/watch?v=VHmQdCTfIzY

The new, air-circulating technique for NGT flushing described here uses the simple observation of air flushed in the side port circulating out the suction port as the key indicator of an empty stomach, as illustrated in Fig. 5. This technique may be used both to maintain the bidirectional patency and function of NGTs placed for obstruction or ileus, as well as NGTs being used for administration of gastric tube feeds. Although several studies evaluate the frequency at which GRV should be checked by NGT flushing [21],volume of GRV at which tube feeds should be held [22, 23], and the size of the NGT [24], the literature fails to address the important question of how to ensure that the measured GRV is the true GRV, in other words, how to ensure bidirectional patency and function of an NGT. The difficulty in ensuring bidirectional patency and function, and therefore knowing the true GRV, is likely the explanation for the poor correlation observed between GRV and aspiration [20, 25, 26].

This study has several limitations. Nonhuman stomachs were used and nonhuman studies are not always amenable to extrapolation to humans. However, pigs stomachs are similar to human stomachs. These pigs were not actually alive at the time of the experiment and following death tissues undergo rigor mortis, which may mitigate the usefulness of the pig model. However, rigor mortis does not begin to occur until 3–4 hours following death, and these experiments took place within the first 30 min following death.

## Conclusion

Only when air flushed into the side port is seen to return out through the main, suctioning port may the tube be deemed properly functional, and accordingly then may GRV be assessed. This technique should be widely taught and adopted to minimize risk of NGT dysfunction and aspiration.
